# Targeted Propolis-Loaded Poly (Butyl) Cyanoacrylate Nanoparticles: An Alternative Drug Delivery Tool for the Treatment of Cryptococcal Meningitis

**DOI:** 10.3389/fphar.2021.723727

**Published:** 2021-08-20

**Authors:** Patcharin Thammasit, Chayada Sitthidet Tharinjaroen, Yingmanee Tragoolpua, Volker Rickerts, Radostina Georgieva, Hans Bäumler, Khajornsak Tragoolpua

**Affiliations:** ^1^ Division of Clinical Microbiology, Department of Medical Technology, Faculty of Associated Medical Sciences, Chiang Mai University, Chiang Mai, Thailand; ^2^ Charité-Universitätsmedizin Berlin, Institute of Transfusion Medicine, Berlin, Germany; ^3^ Department of Microbiology, Faculty of Medicine, Chiang Mai University, Chiang Mai, Thailand; ^4^ Infectious Disease Research Unit (IDRU), Division of Clinical Microbiology, Department of Medical Technology, Faculty of Associated Medical Sciences, Chiang Mai University, Chiang Mai, Thailand; ^5^ Department of Biology, Faculty of Science, Chiang Mai University, Chiang Mai, Thailand; ^6^ Mycotic and Parasitic Agents and Mycobacteria, Department of Infectious Diseases, Robert Koch Institute, Berlin, Germany; ^7^ Department of Medical Physics, Biophysics and Radiology, Medical Faculty, Trakia University, Stara Zagora, Bulgaria

**Keywords:** propolis, poly (n-butyl cyanoacrylate) nanoparticles, blood-brain barrier, *Cryptococcus neoformans*, *in vivo* model

## Abstract

In this study, we describe a nano-carrier system for propolis that is able to cross an *in vitro* model of the blood-brain barrier (BBB) and effectively reduce the virulence of *Cryptococcus neoformans* in animal models. Antimicrobial properties of propolis have been widely studied. However, propolis applications are limited by its low water solubility and poor bioavailability. Therefore, we recently formulated novel poly (n-butyl cyanoacrylate) nanoparticles (PBCA-NP) containing propolis. PBCA-NP are biocompatible, biodegradable and have been shown to effectively cross the BBB using apolipoprotein E (ApoE) as a ligand. Prepared nanoparticles were characterized for particle size, zeta potential, propolis entrapment efficiency and *in vitro* release. Additionally, the PBCA-NP were functionalized with polysorbate 80, which then specifically adsorbs ApoE. Using an *in vitro* BBB model of human brain microvascular endothelial cells hCMEC/D3, it was shown that fluorescence labelled ApoE-functionalized PBCA-NP were internalized by the cells and translocated across the cell monolayer. Propolis-loaded PBCA-NP had *in vitro*, antifungal activity against *C. neoformans*, which causes meningitis. To utilize the invertebrate model, *Galleria mellonella* larvae were infected with *C. neoformans* and treated with propolis-loaded PBCA-NP. The larvae exhibited normal behavior in toxicity testing, and treatment with propolis-loaded PBCA-NP increased survival in the *C. neoformans*-infected larvae group. In addition, following cryptococcal infection and then 7 days of treatment, the tissue fungal burden of mice treated with propolis-loaded PBCA-NP was significantly lower than control groups. Therefore, our ApoE-functionalized propolis-loaded PBCA-NP can be deemed as a potential targeted nanoparticle in the therapeutic treatment of cerebral cryptococcosis.

## Introduction

Cryptococcal meningitis remains a common cause of infectious morbidity and mortality especially among HIV-positive patients living in resource-poor settings, particularly in Southeast Asia and Africa. Current guidelines for induction therapy of AIDS-related cryptococcal meningitis recommend the combination of intravenous amphotericin B deoxycholate (0.7–1 mg/kg/day) and oral 5-fluorocytosine (5-FC, 100 mg/kg/day) for at least 2 weeks (Saag et al., 2000). Amphotericin B is considered a membrane-active agent, as it has been shown to bind to ergosterol in the fungal membrane and disrupt the integrity, causing leakage of small ions and other cellular components (Kagan et al., 2012). However, toxic and therapeutic effects can occur simultaneously, mainly through channel formation in the membrane of kidney cells causing nephrotoxicity. Thus, the toxicity concern of amphotericin B has prompted the search for natural therapeutic alternatives.

Propolis, a natural brown resinous mixture produced by honeybees, has been shown to exert a variety of biological and pharmacological properties. It has long been used for the prevention and treatment of a variety of diseases due to its antimicrobial, antioxidant, anti-inflammatory, and immune-strengthening properties (Sforcin, 2016; Cornara et al., 2017). Ethanolic extracts of propolis have been found to be effective against a broad range of bacteria, viruses, and fungi. These antimicrobial activities have been related with contained phenolics, flavonoids and derivatives of caffeic acid. The chemical composition depends on the location, climate, time of collection and the type of bee species (Uzel et al., 2005). According to the geographical and botanical origins of propolis samples, flavonoids, phenolic acids and their esters, terpenes, as well as fatty acids, are considered the major classes of typical chemical compounds. The detailed chemical profile, nevertheless, varies according to the plants surrounding the beehive. Several studies concluded that Asian, African, and European propolis contains predominantly phenolics and flavonoids such as galangin, pinocembrin, apigenin, pinobanksin, quercetin, cinnamic acid and its esters, kaempferol, chrysin, caffeic acid, *p*-coumaric acid, aromatic acids and their esters (Bankova, 2005; Huang et al., 2014). The HPLC analysis of our propolis extract resulted in concordance with these findings (Iadnut et al., 2019). Gallic acid, quercitin, pinocembrin, chrysin, and galangin were found in the ethanol extract of our propolis samples that are responsible for its biological and pharmaceutical properties (Torres et al., 2018). We found that the high efficacy of sub-MIC (ranging from 0.5 to 0.125 mg/ml) of ethanol extract of propolis possessed the ability to decrease cryptococcal virulence factors (Thammasit et al., 2018). Although extraction with ethanol is suitable for revealing the mode of propolis against fungal virulence factors, it has some limitations of application because of its scarce solubility in water.

In recent years, many specific pharmaceutical approaches such as nanotechnology have been developed to improve the bioavailability of drugs and natural products with low water solubility. As one promising delivery system with improved bioavailability, polymeric nanoparticles based on PBCA have attracted considerable attention. Butyl cyanoacrylate (BCA) is an intermediate-length cyanoacrylate and is a main component of the broadly used medical cyanoacrylate glues (Oowaki et al., 2000) and PBCA is a biodegradable, biocompatible, and bioadhesive polymer. Therefore, PBCA-nanoparticles (PBCA-NP) are biocompatible, biodegradable and their advantages include easy fabrication, functionalization and high entrapment efficiency of poorly soluble substances (Graf et al., 2009; Nicolas and Couvreur, 2009). PBCA-NP have been adopted as a drug delivery system in cancer chemotherapy (Evangelatov et al., 2016), as well as for drug delivery through the BBB (Ramge et al., 2000; Kreuter, 2001; Azarmi et al., 2006; Weiss et al., 2008). There have been several reports of PBCA-NP coated with polysorbate 80 that could significantly improve the delivery to the brain of anticancer agents (Gulyaev et al., 1999), antifungal drugs (Mura et al., 1999; Kuo and Chen, 2006), peptides (Kreuter et al., 1995), antibiotics (Page-Clisson et al., 1998). PBCA-NP were loaded with dalargin (a hexapeptide analog, which produces CNS analgesia) and coated with polysorbate 80. These nanoparticles were delivered intravenously in the C57BL/6J mice model achieving therapeutic drug concentrations within the CNS, which was observed by antinociceptive activity of the animals due to strong analgesic effect of the dalargin-loaded PBCA-NP (Kreuter et al., 2002; Kim et al., 2009). The mechanisms behind the ability of polysorbate 80 coated PBCA-NP to cross the BBB, is not fully understood. The most likely mechanism appears to be LDL-receptor-mediated endocytosis in the microvascular endothelium of the brain and transcytosis. It was suggested that ApoE, an exchangeable apolipoprotein that is responsible for the transport of cholesterol is involved in this process adsorbing selectively ad on the polysorbate 80 functionalized surface of the PBCA-NP after their injection into the blood (Kreuter et al., 2002).

The aim of our work presented here was to formulate a nano-carrier system for propolis that is able to cross the BBB, deliver its load to the CNS and effectively reduce the virulence of *C. neoformans*. Using an anionic emulsion polymerization method, we fabricated biodegradable propolis loaded PBCA-NP functionalized with polysorbate 80 and ApoE. The particles were characterized concerning their size, polydispersity index (PDI), zeta potential, propolis encapsulation efficiency, drug-release behavior and stability. An *in vitro* model of the BBB, constituted by human cerebral microvascular endothelial cell line (hCMEC/D3) was established and used to evaluate the ability of propolis-loaded PBCA to pass through this barrier and their effectiveness inhibiting *C. neoformans* to cross the barrier. Invertebrate infection model based on *Galleria mellonella* larvae (Champion et al., 2018) and animal mice model were further applied for the approval of the therapeutic potential of propolis-loaded PBCA-NP.

## Materials and Methods

### Materials

Propolis powder was kindly provided by Bee Product Industry Co., Ltd., Lamphun, Thailand. The stock solution was prepared in dimethyl sulfoxide (DMSO). The chemical characteristic of propolis was carried out in a previous study (Iadnut et al., 2019). n-butyl cyanoacrylate (n-BCA) monomer was received as a gift sample from Tong Shen Enterprise Co., Ltd., Taiwan. Poloxamer 188 and polysorbate 80 were obtained from Sigma Chemical Co. (St. Louis, MO). All other chemicals and reagents used in this study were of analytical grade.

### Preparation of Propolis-Loaded poly(n-Butyl Cyanoacrylate) Nanoparticles (PBCA-NP)

**FIGURE 1:**
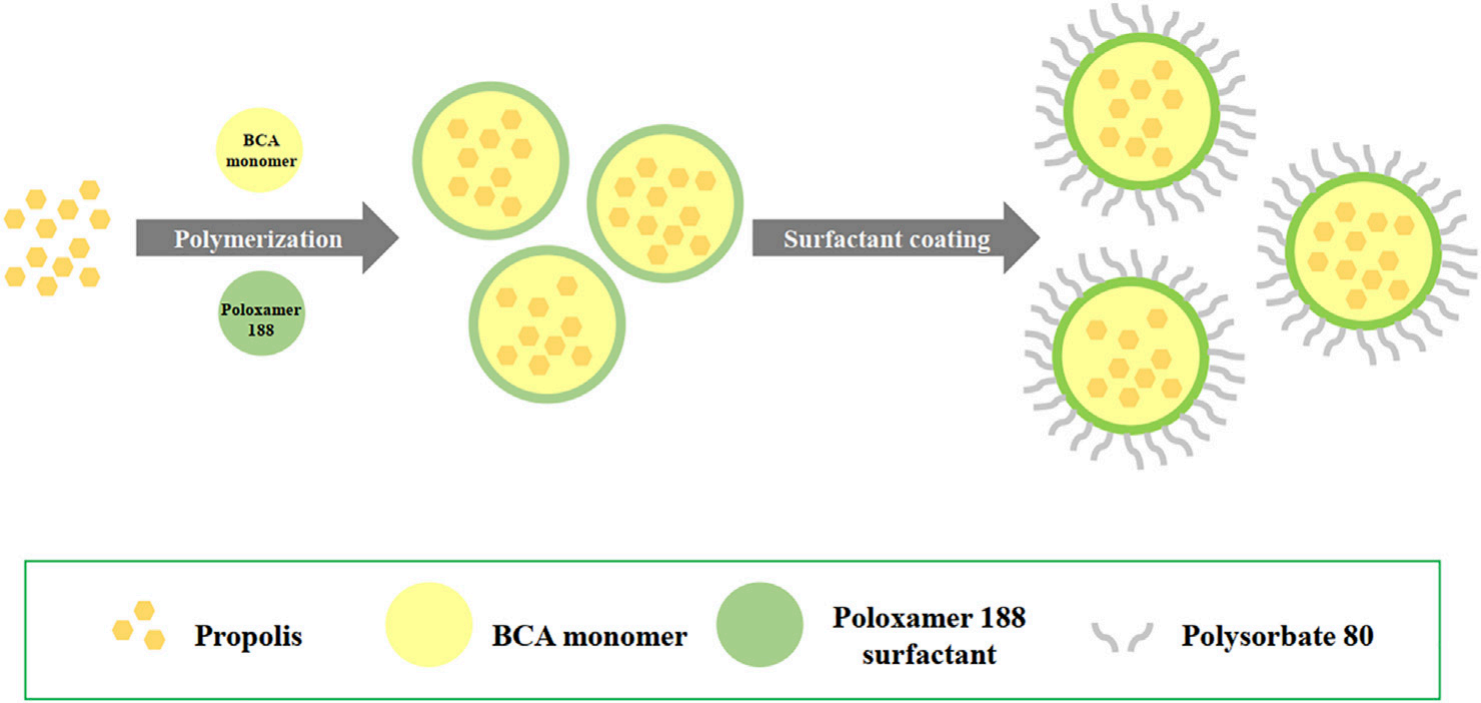
Schematics showing the preparation of propolis-loaded PBCA-NP by anionic emulsion polymerization.

As shown in Figure 1, propolis-loaded PBCA-NP were prepared by *in situ* anionic emulsion polymerization method. Poloxamer 188 (1%) was dissolved in 20 ml of 0.01 N of HCl and propolis (50 mg; final concentration 2.5 mg/ml) was diluted in 1 ml of absolute ethanol and then added to acidic aqueous medium. Then 1% of n-BCA monomer was slowly added to the aqueous phase under stirring on magnetic stirrer, and the monomer was allowed to polymerize. After 4 h, the nanoparticle suspension was neutralized to pH 7.0 using 0.1 N NaOH and stirring was continued for 1 h to complete the polymerization. The dispersion was incubated in 1% Polysorbate 80 solution for 30 min under stirring, and centrifuged at 18,000 *g* for 1 h. In the cell culture experiments, 20 μg/ml of ApoE (Sigma-Aldrich, MO) was freshly conjugated to the PBCA-NP by stirring for 1 h at 37°C prior to the experiments. In some experiments, the PBCA-NP were labelled *in situ* with a fluorescent dye, Nile red at 10 μg/ml for the final concentration.

### Physicochemical Characterization of PBCA-NP

#### Size Distribution and Zeta Potential Measurements

The particle sizes and PDI of the nanoparticles were measured by Dynamic Light Scattering (DLS) using a Malvern instrument (Nano Sizer ZS90; Malvern Instruments, Inc., Malvern, United Kingdom) at 25°C and 90° angle of detection. Prior to measurement, the suspensions were dispersed in PBS at an appropriate concentration for better measurement. The surface charges (zeta potentials) were investigated by Laser Doppler Velocimetry using the same instrument. The samples were analyzed in triplicates with three readings per nanoparticle sample. Values of the particle sizes and zeta potentials are presented as mean ± SD from three replicate samples.

#### Particulate Morphology

A scanning electron microscope (SEM) was used to verify the uniformity of particle shape and size. Nanoparticle suspension was dropped onto aluminum SEM stubs with double-sided sticky tape and dried at room temperature. The SEM stubs were then coated with 150 Å gold for 2 min by the SPI Module gold sputter coater. The surface morphology of the samples was finally observed under SEM (JEOL ltd., Japan) operated at 15-keV pulse at different resolutions.

#### Fourier Transform Infrared (FTIR) Spectroscopy

FTIR studies were carried out to investigate the functional groups of propolis after loading to PBCA polymer. FTIR spectra of propolis, propolis-loaded, and empty PBCA-NP were recorded on Shimadzu Fourier Transform Infrared Spectrophotometer (Shimadzu 8400S, Japan). Test samples were mixed with 1,000 mg of potassium bromide (KBr) powder, pressed into a disk and scanned from 400 to 4,000 cm^−1^.

#### Determination of Propolis Encapsulation Efficiency

To determine the total amount of propolis associated with the particles, the nanoparticles were dissolved in tetrahydrofuran (THF). The solutions were stirred for 4 h to dissolve the particles, and the amount of propolis was spectroscopically determined measuring its adsorption at 267 nm in the Supplementary Figure S1 (U-2800, Hitachi, Japan). The percentage of drug entrapment efficiency (%EE) was calculated using the following formula:

$$\text{Direct method:}\%EE=\left(\frac{AR}{AI}\right)\times 100$$

Where: %EE is entrapment efficiency, AR is the amount of propolis released from the PBCA-NP and AI is the amount of propolis initially taken to prepare the nanoparticles.

The amount of propolis entrapped in the nanoparticles was also indirectly determined as the difference between the amounts of total propolis initially applied (AI) and the amount of propolis, which was determined in the supernatant after particle preparation and after each washing step (AS). The propolis concentration in the supernatants was also measured using UV–Vis spectroscopy as described above. The %EE was calculated according to the following equation:

Indirect method:%EE=(AI−AS)×100/AI

#### *In vitro* Release Studies

Nanoparticle formulations were dried using lyophilization. The sample dry weight of formulations equivalent to 1 mg of propolis-loaded PBCA-NP was suspended into separate tubes containing 100 µL of PBS with pH 4.0, pH 5.5, and pH 7.4) and incubated on a rotator at 37°C. Then, the medium was withdrawn at each time point (0, 1, 2, 3, 6, 12, 24, and 48 h), and replaced with an equal volume of fresh medium buffer. The amount of released propolis from the PBCA-NP was analyzed by UV–Vis spectroscopy at 267 nm (U-2800, Hitachi, Japan). The cumulative propolis release at different times was calculated as percentage of the total encapsulated amount. The experiments were performed in triplicate, and the obtained data were kinetically analyzed to determine the pattern of drug release.

#### Stability Studies

PBCA-NP in lyophilized powder form were stored at 4°C in the dark condition and then evaluated by measuring size and PDI by means of dynamic light scattering for 1 month, after appropriate dilution with physiological phosphate buffer.

#### Determination of anti-Cryptococcal Activity of Propolis-Loaded PBCA-NP

*Cryptococcus neoformans* H99 (kindly provided by Assoc. Prof. Dr. Pojana Sriburee from the Faculty of Medicine, Chiang Mai University, Thailand) was cultured in Sabouraud Dextrose Agar (SDA) at 37°C for 72 h and prepared by diluting the yeast cells with cell culture medium, which was determined using a hemocytometer and microscopic observation. *C. neoformans* H99 cells were seeded in tissue culture plates of 96 wells at a concentration of 4 × 10^5^ cells per well. After 24 h of incubation, five concentrations of a series of 10-fold dilutions (10^8^–10^12^ particles; 0.66–6,600 μg/ml propolis concentrations) of the propolis-loaded PBCA-NP (or empty PBCA-NP) were added to the wells containing yeast cells. The number of nanoparticles was estimated assuming spherical geometry calculation in the colloidal solution. After 3 h of incubation at 37°C, the medium was removed and 20 µL of 5 mg/ml MTT solution was added to the wells. Four hours later, the MTT formazan product was dissolved in DMSO and the absorbance was measured using a microplate reader at 540 and 630 nm.

### *In vitro* Model

#### Culture of hCMEC/D3 Cells

Collagen gels were prepared at a collagen concentration of 150 μg/ml collagen type I, rat tail (Merck Millipore, Darmstadt, Germany) in PBS. To allow collagen gel formation, the flask was incubated at 37°C for 1 h in a humidified atmosphere.

Immortalized human cerebral microvascular endothelial hCMEC/D3 cells were maintained in 75 cm^3^ flasks precoated with collagen in EndoGRO Basal Medium (Merck Millipore, Darmstadt, Germany) supplemented with 1 ng/ml human fibroblast growth factor basic protein (FGF-2) (Merck Millipore, Darmstadt, Germany), 0.2% EndoGRO-LS supplement, 5 ng/ml recombinant human epidermal growth factor, 50 μg/ml ascorbic acid, 10 mM l-glutamine, 1 μg/ml hydrocortisone hemisuccinate, 0.75 U/mL heparin sulfate, 2% (v/v) fetal bovine serum (FBS), 100 units/mL of penicillin and 100 μg/ml streptomycin. The cells are cultivated at 37°C in a humidified atmosphere with 5% CO_2_.

#### Cellular Uptake Experiment

The optimum concentration of PBCA-NP was determined by MTT assay according to the previous protocol (Kong et al., 2011). Cells (1 × 10^5^ cells/well) were cultivated in tissue culture plates of 24 wells overnight to allow cell attachment. The culture medium was replaced by medium containing nanoparticles; Nile red-labelled propolis-loaded, and Nile red-labelled empty PBCA-NP, respectively, and cells were incubated for 3 h at 37°C. After incubation, the medium was removed, and cells were thoroughly washed thrice with PBS to remove any excessive nanoparticles. To track the endocytic pathway, the cells were pre-labeled 30 min beforehand with 20 nM LysoTracker Green, a green fluorescent dye with excitation/emission maxima ∽504/511 nm (Thermo Fisher Scientific, MA), lysosome marker. Incubation at 37°C was performed with protection from exposure to light. The cellular uptake was detected by flow cytometry (BD FACSCanto II, Becton Dickinson, NJ) and quantified according to previous study (Maussang et al., 2016), detecting Nile red signal in PE channel. In addition, the uptake was finally visualized by confocal laser scanning microscopy (CLSM) (LSM 510 META, Carl Zeiss Micro Imaging GmbH, Jena, Germany).

#### Endothelial Transcytosis Experiment

**FIGURE 2:**
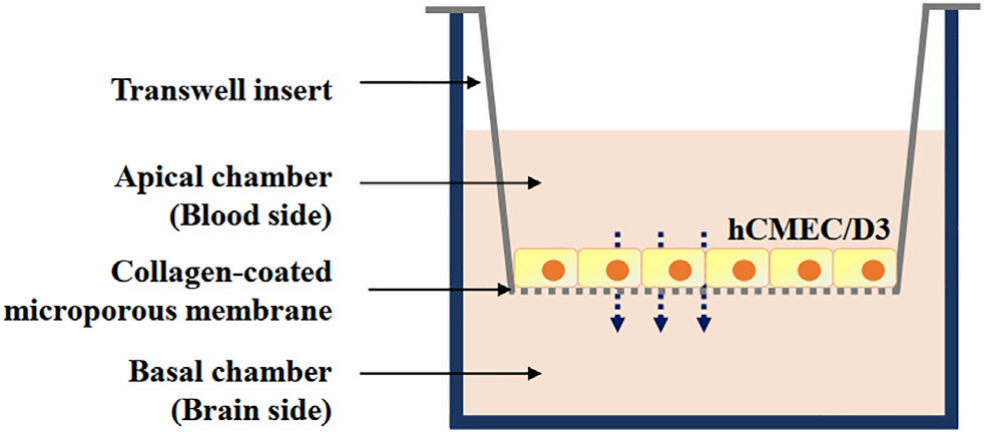
Schematic representation of blood-brain barrier *in vitro* model constructed using immortalized human brain capillary endothelial cell line (hCMEC/D3) cell lines grown on Transwell inserts: a monoculture.

For the *in vitro* blood–brain barrier (BBB) model*,* a monolayer of hCMEC/D3 brain microvascular endothelial cells was used. It closely mimics the *in vivo* phenotype, is easy reproducible, and highly desirable for drug screening and permeation studies (Bagchi et al., 2019). Transwell 24-well tissue culture plates with individual plate wells (lower chamber) fitted with inserts (upper chamber) containing 8 µm pore-size collagen-coated polycarbonate membranes (Corning, NY) were used (Figure 2). The cells (passage 26–35) were seeded at a density of 1 × 10^5^ cells/cm^2^ into the inserts (upper chamber) and grown for 5 days in 1 ml of culture medium. Medium was changed every other day and 24 h before the assay, the medium was then changed to a 1/2 dilution in order to reduce growth factors and to promote cell differentiation and tight-barrier formation.

The cell barrier integrity was verified prior to performing endothelial permeability experiments by means of *trans*-endothelial electrical resistance (TEER), using a Millicell ERS-2 Voltohmmeter (Merck Millipore, Darmstadt, Germany). TEER readings of hCMEC/D3 cultures were taken at several time points after seeding in Transwell inserts, and maximum resistance typically occurred after 5 days of culture. Background TEER values of resistance across inserts without hCMEC/D3 cells were subtracted. Monolayers of hCMEC/D3 cells, with TEER values approximately 40 Ω cm^2^ were used in this study (Helms et al., 2016).

Briefly, hCMEC/D3 cell monolayers were washed once with prewarmed Hank’s Balanced Salt Solution (HBSS). Subsequently, 500 μL of Nile red-labelled propolis-loaded or empty PBCA-NP diluted in EBM-2 were added apically to the cells and incubated at 37°C for 3, 6, and 24 h. After the incubation period, the medium was collected, and the cells were washed with 500 μL prewarmed HBSS to collect residual PBCA-NP (total volume of apical fraction: 1 ml). The collagen gels were digested in 200 μL 0.25% (w/v) collagenase D (Roche, Basel, Switzerland) in HBSS for 90 min at 37°C. The cells were pelleted by centrifugation at 200 *g* for 5 min. The supernatant was collected and mixed with 400 μL EBM-2 medium (total volume of basolateral fraction: 1 ml). The hCMEC/D3 cell pellet was soaked in 500 μL of ultrapure water for 10 min, and subsequently mixed with 500 μL of EBM-2 medium (total volume of cellular fraction: 1 ml).

The fluorescence intensities (FI) in the apical, cellular, and basolateral fractions were measured in triplicate using black, flat-bottomed microplates and a BioTek Cytation 3-cell imaging multi-mode reader (Fisher Scientific, NH) with excitation and emission at 540 and 630 nm, respectively. The fluorescence in the distinct apical, cellular, and basolateral fractions without PBCA-NP, i.e., background fluorescence, was subtracted from the measured intensity values. The FI of apical, cellular and basolateral fractions are related with the determination of particles concentrations that pass the BBB. In order to determine the permeability values across blank Transwell inserts, the FI in the inserts without seeded cells were evaluated in triplicate. The %NP translocation was calculated based on the Nile red fluorescence intensity according to the following equation:

$$\text{\%Translocation}=\frac{\text{FI of the basolateral fraction}}{\text{FI of }\left(\text{apical}+\text{cellular}+\text{basolateral fractions}\right)}\times 100$$

#### Effect of Propolis-Loaded Nanoparticles Against Cryptococcal Infection in BBB Model

The hCMEC/D3 cells (10^5^ cells per insert) were grown in the upper chamber for 5 days. *C. neoformans* H99 (5 × 10^5^ cells/well) were added to the upper chamber to give a multiplicity of infection (M.O.I.) of 5: 1 (yeast to hCMEC/D3 cells ratio) and incubated for 21 h to allow the yeasts pass through the membrane. After that, propolis-loaded or empty PBCA-NP (10^11^ particles/well) were added and the cells were further incubated at 37°C with 5% CO_2_ for 24 h. The number of cryptococcal cells that crossed the endothelial cell barrier was quantified by the number of colony forming units (C.F.U.) recovered by serial dilution, from 100 µL aliquots withdrawn from the lower Transwell chambers and plated on SDA.

## Invertebrate Model

*Galleria mellonella* larvae have successfully been used in human infectious disease studies (Fuchs et al., 2010). In our study, last instar larvae of *G. mellonella* weighing 250–350 mg were used to screen for toxicity of propolis loaded PBCA-NP and antifungal activity against *C. neoformans strain* H99. Groups of 20 larvae were used per experimental group. Each experiment included three negative control groups: without manipulation (no touch group), with injection of physiologic saline and with injection of heat killed *C. neoformans* H99 (65°C, 30 min). Before inoculation, larvae were disinfected by gentle swabbing with 70% ethanol. Inocula (20 µL) were injected using 30G insulin syringe needles (Omnican, Braun, Melsungen) into the last left or right proleg. Larvae were housed in Petri dishes containing wood chips and were incubated at 37°C. Larvae were checked daily for survival, defined as movement upon stimulation. Pupated larvae were excluded on the day of pupation.

Acute toxicity was assessed by injection of 20 µL inocula of empty and propolis-loaded PBCA-NP suspensions. The toxicity of the particles was evaluated by comparing survival curves of the “no touch” and physiologic saline injected groups with the NP injected groups.

For determination of the antifungal activity of propolis-loaded PBCA-NP, larvae were infected with 2 × 10^6^ *C. neoformans* H99 (20 µL into the last left proleg). After 4 h, propolis-loaded or empty PBCA-NP were injected to the last right pro-leg. The dose of propolis applied with the particles corresponded to the previously determined IC50 (0.7 μg/ml; 20 µL/larvae). Larvae infected with cryptococci (untreated) were the infection positive control. The antifungal activity of propolis-loaded PBCA-NP was assessed by comparing the survival curves of non-treated infected larvae, larvae treated with empty PBCA-NP and larvae treated with propolis-loaded PBCA-NP.

Survival curves were generated in Graphpad prism eight and differences between survival curves were evaluated by log-rank test.

### *In vivo* Model With Intravenous Injection

#### Mice Model Preparation

Healthy female BALB/c mice weighing 20 ± 5 g (provided by Nomura Siam International Co. Ltd., Bangkok, Thailand) were used in the biodistribution and therapeutic studies. All mice were housed in aero mouse individually ventilated cage with pathogen-free environment and received sterilized food and water *ad libitum*. Animal care and use were performed in strict accordance with an animal use protocol reviewed and approved by the Laboratory Animal Center (LAC), Chiang Mai University (permit number 2561/MC-0002) in accordance with the Guide for the Care and Use of Laboratory Animals of US National Research Council 2010.

According to the study of *Cryptococcus* in murine model, antifungal treatment was previously described elsewhere (Xu et al., 2011; Thompson et al., 2012). Animal studies were divided into four groups: untreated controls, amphotericin B treatment and groups treated with either empty or propolis-loaded PBCA-NP by i. v. injection. Because of the generally virulence observed with *C. neoformans*, anti-cryptococcal treatment was initiated 1 day after inoculation and continued until day 8 in fungal burden studies (n = 12 mice per group). Injection was delivered as a 50 µL volume through a 27-gauge needle fastened to a tuberculin syringe. Before 24 h starting the experiment, BALB/c mice were i. p. administered with 250 mg/kg of cyclophosphamide (Endoxan, Baxter, IL) to induce immunosuppression. After 24 h of administration, BALB/c mice were then inoculated with the *C. neoformans* H99 suspension (concentration 1.4 × 10^7^ CFU/ml, dose 7 × 10^5^ CFU/50 µL/20 g) through i. v. injection.

The accurate determination of dose when studying nanoparticle treatment is essential for quantitative particle toxicology and therapeutics. Dose of nanoparticles was calculated according to the volume of inspiration based on the animal’s body weight. Stock suspensions of the nanoparticles [empty (17.25 mg/50 µL/20 g) and propolis-loaded PBCA NP (30.75 mg/50 µL/20 g)] were prepared in 50 µL of 0.9% sodium chloride and injected *via* i. v. injection. Amphotericin B was also prepared with 3 mg in 50 µL of 0.9% sodium chloride per mouse.

#### Tissue Fungal Burden

Twenty-four hours after the last injection (day 8), the mice were sacrificed by intraperitoneal pentobarbital euthanasia technique and the brains, lungs, and kidneys were dissected, weighed, and homogenized with sterile saline. The homogenate was serially diluted with sterile saline, then 100 µL of the suspension was inoculated onto SDA plates and incubated at 37°C for 48 h and counted the number of colonies (C.F.U/Gram tissue).

#### Histopathology

Brain tissues were also placed into phosphate-buffered formalin. The tissues were processed and embedded in paraffin wax and 5 mm sections were made. The sections were then stained with hematoxylin and eosin (H&E) and viewed by light microscopy (Vance, 1961).

### Statistical Analysis

All the aforementioned experiments were presented as mean ± SD calculated over at least three data points. The differences between experimental groups were analyzed by one-way analysis of variance with a post hoc Tukey comparison test. The comparisons of survival curves by the Kaplan-Meier method were investigated using the log-rank test. A value of *p* ≤ 0.05 was considered to be statistically significant.

## Results

### Physicochemical Characteristics of PBCA-NP

**FIGURE 3:**
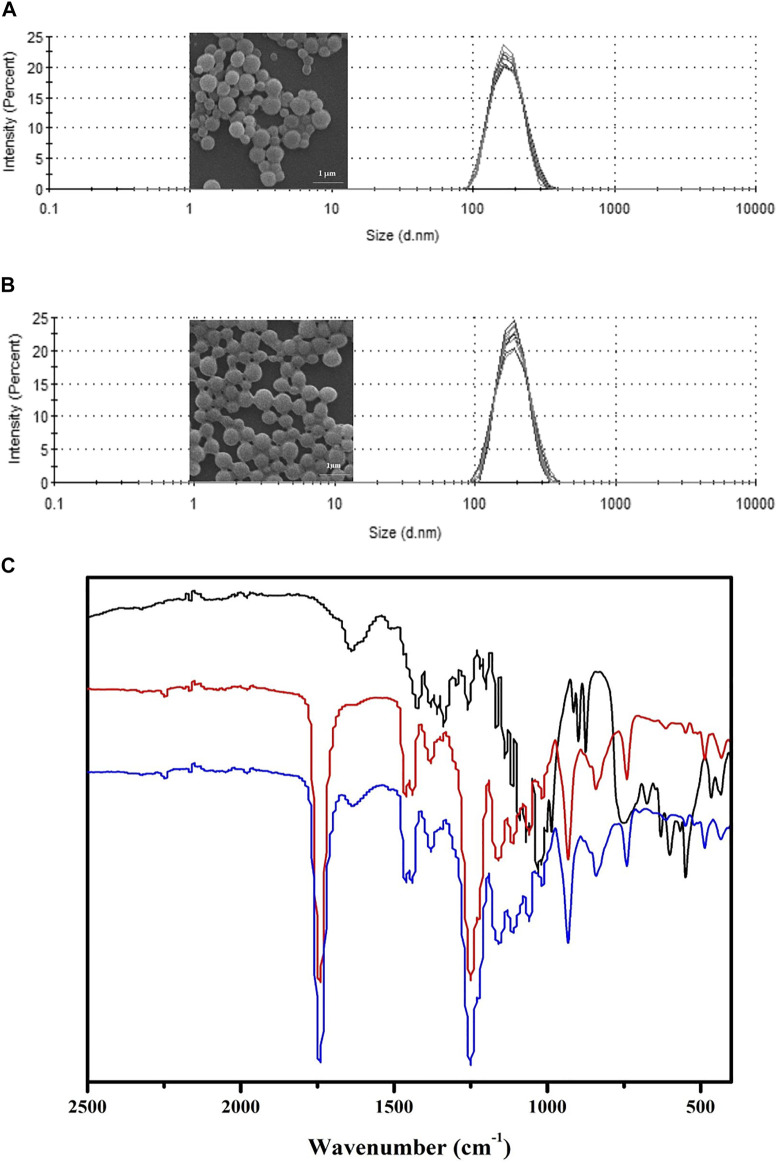
Characterization of propolis-loaded and empty PBCA-NP. Particle size distribution and SEM images of propolis-loaded **(A)** and empty **(B)** PBCA-NP. FTIR spectra **(C)** of propolis extract (black line), empty PBCA-NP (red line), and propolis-loaded PBCA-NP (blue line) demonstrating absorption band at 1,638 cm^−1^ region.

PBCA-NP containing propolis were prepared, as described, using an *in situ* anionic emulsion polymerization method. The full characterization in terms of dimension, PDI, zeta potential, and propolis entrapment efficiency (%EE) was carried out and results are presented in Figures 3A,B. The obtained nanoparticles were spherical in shape and displayed a monomodal size distribution, which is also confirmed by the SEM observation. Table 1 demonstrated propolis -loaded and empty PBCA-NP exhibited a mean diameter of 194.8 ± 2.135 and 196.4 ± 2.178 nm, respectively, as measured by DLS. Zeta potential measurements at physiological ionic strengths in phosphate buffered solution (conductivity 16.7–18.1 mS/cm) showed negative zeta potential of 8–9 mV for both of the propolis-loaded and empty PBCA-NP. The drug encapsulation efficiency was higher than 88%.

**TABLE 1:** The averaged data on particle size, polydispersity index (PDI), zeta potential, and propolis encapsulation efficiency (%EE). n = 3.

Sample	Average size (nm)	PDI	Zeta potential (mV)	%EE	
				Direct	Indirect
Propolis-loaded PBCA-NP	194.8 ± 2.1	0.083	−8.78 ± 1.2	88.84 ± 0.9	91.31 ± 0.8
Empty PBCA-NP	196.4 ± 2.2	0.086	−8.14 ± 1.4	ND	ND

To assess the presence of propolis extract in PBCA-NP, FTIR spectroscopy was used to investigate the major functional groups of propolis extract, empty PBCA-NP, and propolis-loaded PBCA-NP, as shown in Figure 3C. Overall, the FTIR absorbance profiles of propolis-loaded PBCA-NP are very similar to those of PBCA-NP. Propolis-loaded PBCA-NP exhibited carbonyl stretching at 1,750 cm^−1^ which corresponds to the ester functional group of PBCA-NP. Furthermore, carbonyl stretching of the ester functional group at 1,638 cm^−1^ of propolis extract was observed in propolis-loaded PBCA-NP. Therefore, FTIR results suggest that the existence of propolis and PBCA-NP in propolis-loaded PBCA-NP.

**FIGURE 4:**
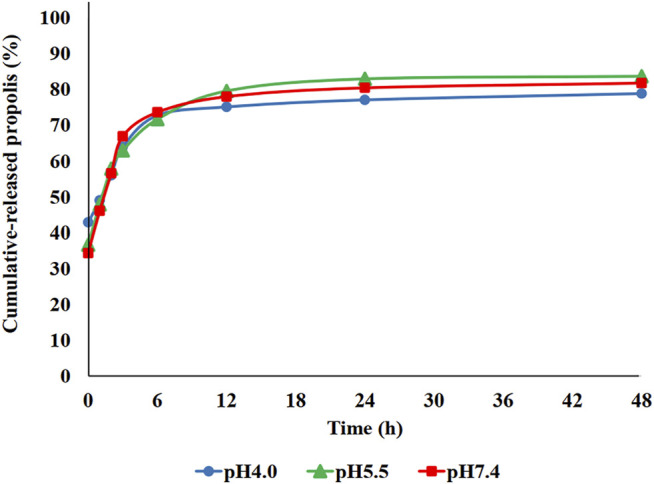
*In vitro* cumulative release profile of propolis-loaded PBCA-NP in PBS at three different pH values as measured by solvent displacement. The amount of propolis in the supernatant was determined by measuring the absorbance at 267 nm. n = 3.

*In vitro* release studies were carried out by solvent displacement technique and the obtained cumulative release profiles are reported in Figure 4. The release profile of propolis from the PBCA-NP formulation exhibited biphasic pattern that is characterized by an initial burst, followed by a slower sustained release. More than 70% of propolis was release within 6 h of incubation, and a constant slow drug release was observed up to 48 h.

The stability of the obtained PBCA-NP during storage as lyophilized dry powder at 4°C was examined for 4 weeks. For examination, the size and PDI of the particles was measured every week after recovering them in suspension with physiological phosphate buffer. The results presented in Table 2 confirmed that there was no significant change (*p* > 0.05) and therefore, the empty as well as propolis-loaded PBCA-NP were stable within this period.

**TABLE 2:** Stability of propolis-loaded and empty PBCA-NP during storage as lyophilized dry powder of at 4°C. The size and PDI were determined every week after re-suspension in PBS. n = 3.

Formulations	Week 1		Week 2		Week 3		Week 4	
	Size (nm)	PDI	Size (nm)	PDI	Size (nm)	PDI	Size (nm)	PDI
Propolis-loaded PBCA-NP	187.7 ± 1.35	0.05	188.5 ± 1.34	0.04	181.1 ± 1.31	0.08	187.5 ± 0.88	0.05
Empty PBCA-NP	195.1 ± 1.34	0.08	191.3 ± 1.14	0.08	182.6 ± 1.37	0.10	194.2 ± 1.77	0.09

**FIGURE 5:**
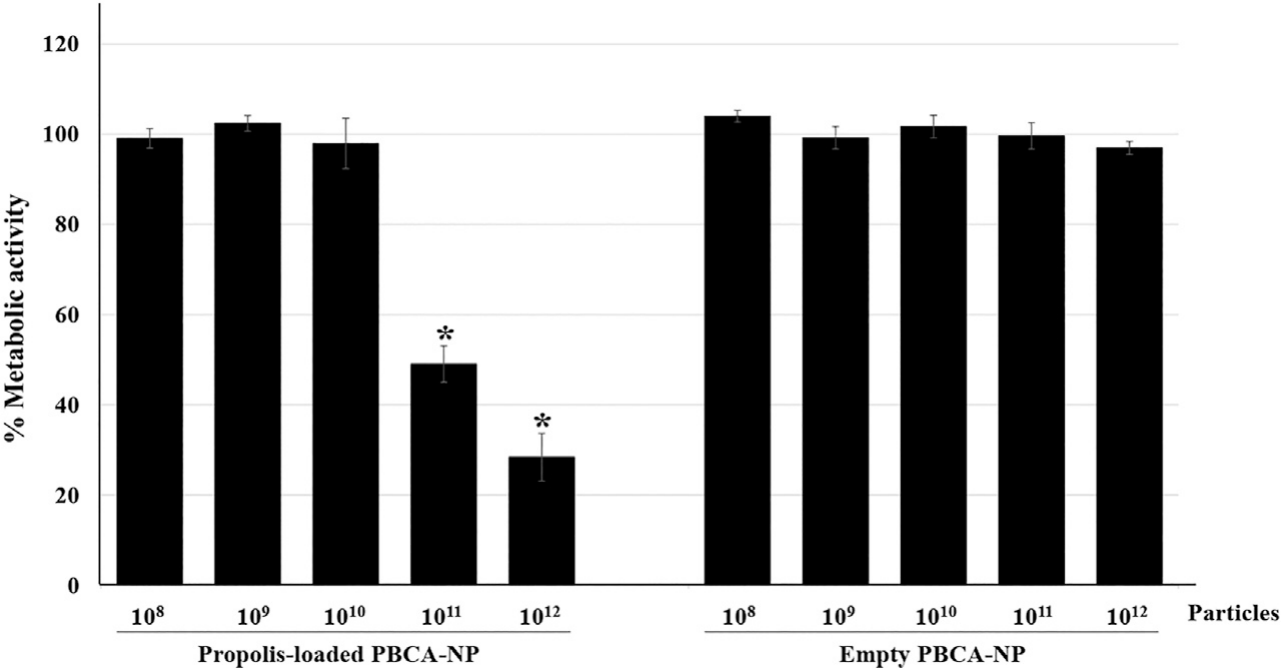
Anti-cryptococcal activity of propolis-loaded PBCA-NP. The metabolic activity of *C. neoformans* H99 was determined by the MTT assay. *C. neoformans* H99 was seeded at a concentration of 4 × 10^5^ cells per well and cultured for 24 h before the addition of empty or propolis loaded PBCA-NP at five different concentrations. The data were represented a percentage of metabolic activity from three independent experiments as an average value and SD. n = 3. “*” *p* ≤ 0.05.

To proof nanoparticle’s property, the anti-mycotic activity of propolis-loaded PBCA-NP against *C. neoformans* H99 was evaluated by MTT assay. As shown in Figure 5, 10^11^–10^12^ propolis-loaded PBCA-NP per well reduced the metabolic activity of *C. neoformans* by more than 50% compared with the control, which was incubated with empty PBCA-NP.

### Cellular Uptake of Propolis-Loaded PBCA-NP

The viability of hCMEC/D3 cells treated with propolis-loaded and empty PBCA-NP was detected by the MTT assay at five concentrations, reflecting that the nanoparticle samples were nontoxic on the hCMEC/D3 cells at the concentrations used (10^5^–10^11^ particles per 10^5^ cells), since the cell survival rates were over 90% (Supplementary Figure S2).

**FIGURE 6:**
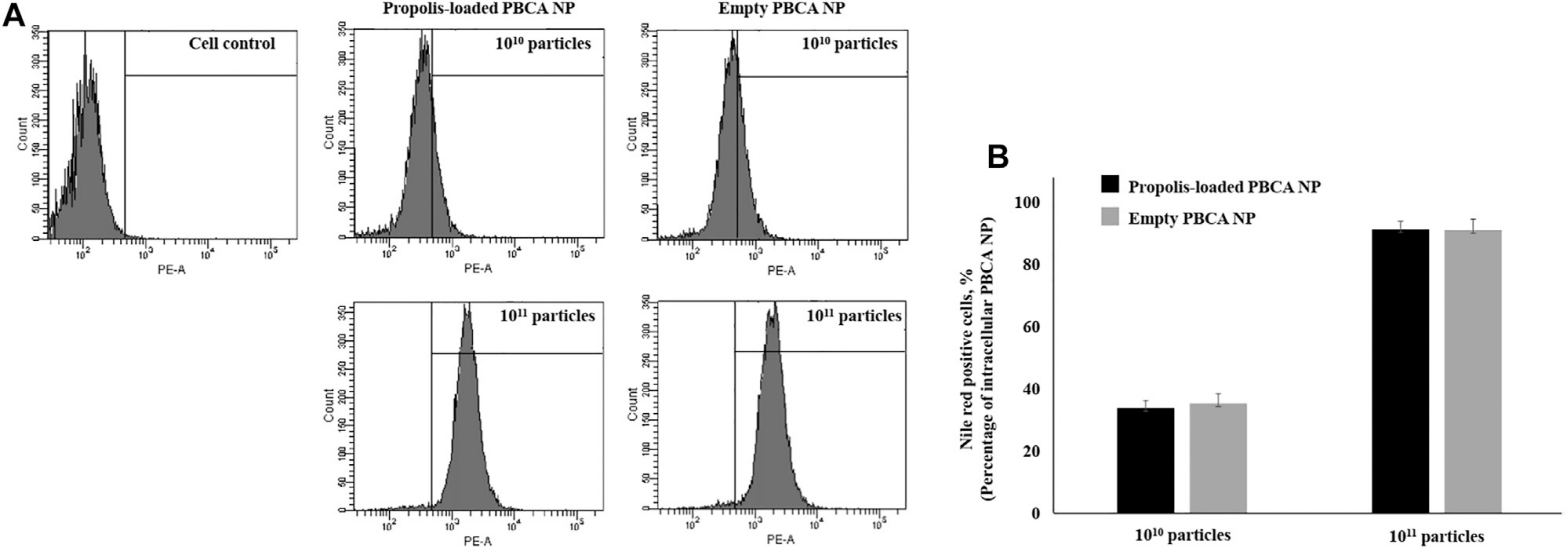
The cellular uptake of PBCA-NP in hCMEC/D3. **(A)** Flow cytometry histograms show cellular uptake of propolis-loaded and empty PBCA-NP along with cell control. Histograms of fluorescence intensity (*x*-axis) *vs.* cell count (*y*-axis) demonstrate uptake percentage by hCMEC/D3. **(B)** The percentage of cells having taken up PBCA NP was quantified as Nile red positive cells. n = 3.

To investigate the percentage of nanoparticle uptake into immortalized human cerebral microvascular endothelial cells hCMEC/D3, cells were incubated with propolis-loaded or empty PBCA-NP and the resulting intracellular fluorescence per cell was measured by flow cytometry. Figure 6A represents the dose-dependent hCMEC/D3 cellular uptake of propolis-loaded and empty PBCA-NP and quantification of PBCA NP taken up inside the cells was demonstrated in Figure 6B. CLSM images of the intracellular localization of ApoE coated propolis-loaded PBCA-NP in hCMEC/D3 cells represented in the Supplementary Figure S3A-D.

### Transcytosis of Propolis-Loaded PBCA-NP Cross an *in vitro* BBB Model

To quantitatively assess the transcytosis of propolis-loaded PBCA-NP in the *in vitro* BBB model, hCMEC/D3 cerebral endothelial cells were grown on 8 μm Transwell membranes in a confluent layer. The integrity of the *in vitro* BBB prior to nanoparticle testing was monitored using TEER measurements. The TEER values showed an increased electrical resistance up to 40 Ω cm^2^ at day 5. Prolonged incubation of hCMEC/D3 cerebral endothelial cells for more than 5 days did not alter the electrical resistance. Therefore, a culture time of 5 days was selected as the optimal time for cell monolayer formation on the Transwell system. The presence of mature, tight junction protein, occludin, in the cell monolayer was also observed using immunofluorescent staining with occludin antibody (ThermoFisher Scientific, 33–1,500). Results demonstrated the expression of occludin throughout the tight junctions of hCMEC/D3 cerebral endothelial cells, as shown in Supplementary Figure S4.

**FIGURE 7:**
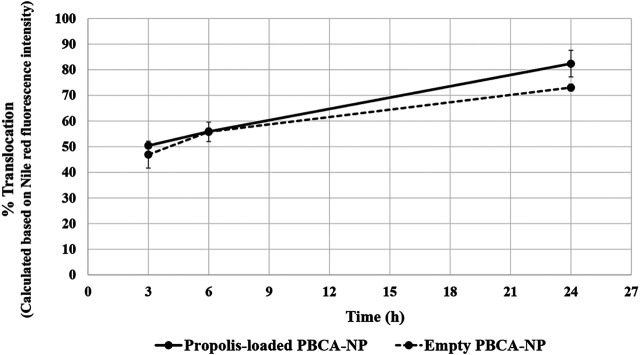
Translocation across an *in vitro* BBB model of propolis-loaded or empty PBCA-NP in the basolateral fraction. The percentage of the NP translocation was calculated based on Nile red fluorescence intensity. n = 3.

For the quantitative analysis of the cellular translocation, it was observed whether the cells were able to exocytose the uptaken PBCA-NP on the basolateral side of the cell layer. The translocation of 10 μg/ml Nile red-labelled propolis-loaded or empty PBCA-NP in the basolateral sides after 3, 6, and 24 h of exposure were analysed measuring the Nile red fluorescence intensity. After 3 h, approx. 50% of the added PBCA-NP were translocated across the barrier, and after 24 h of incubation, approx. 80% of the PBCA-NP passed the cell barrier as shown in Figure 7. In comparison, for Transwell membranes lacking the cell monolayer, the maximum translocation of PBCA-NP was achieved within 3 h (data not shown).

### Effect of Propolis-Loaded PBCA-NP Against *C. neoformans* H99 Strain in BBB Model

**FIGURE 8:**
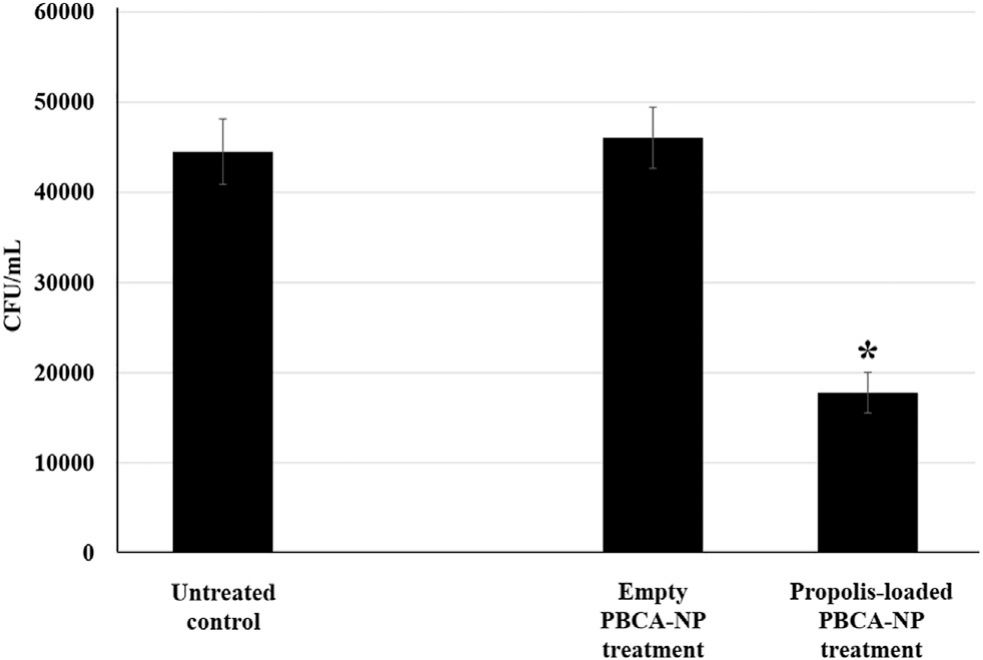
Propolis-loaded PBCA-NP inhibited *C. neoformans* H99 proliferation in BBB model. hCMEC/D3 cells were cultured and infected with *C. neoformans* for 21 h in the apical compartment. After infection, the model was then treated with the indicated number of propolis-loaded or empty PBCA-NP. The number of colonies at the basolateral chamber were determined by the spread plate technique. The data were represented a cell number in term of CFU/mL from three independent experiments as an average value and SD. n = 3. “*” *p* ≤ 0.05.

To determine whether the propolis-loaded PBCA-NP were active against a fungal pathogen in the brain after accumulation and transport through the BBB. In the apical chamber of BBB, the model was infected with five M.O.I. of *C. neoformans* H99 strain and then treated with the indicated number of propolis-loaded and empty PBCA-NP. Our results revealed the cryptococcal cells could migrate across the hCMEC/D3 barrier in 21 h (control) as shown in basal chamber. In Figure 8, we found that the number of colonies significantly decreased in propolis-loaded PBCA-NP-treated well. Meanwhile, there is no statistically significant difference in empty PBCA-NP compared to the untreated control group.

### Effect of Propolis-Loaded PBCA-NP on the Survival of Infected *G. mellonella* Model

**FIGURE 9:**
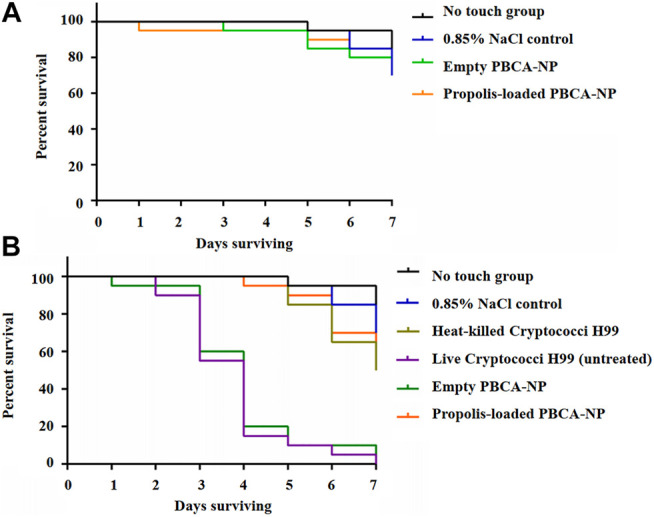
Invertebrate toxicity and survival curves of PBCA-NP towards *G. mellonella* larvae **(A)** Kaplan–Meier survival curves of *G. mellonella* larvae represent *in vivo* toxicity testing of PBCA-NP. Randomly chosen *G. mellonella* larvae were injected with propolis-loaded or empty PBCA-NP. Two different controls were used: no touch larvae and larvae injected with 0.85% NaCl (**B)** Kaplan–Meier survival curves of *G. mellonella* larvae after infection with *C. neoformans*. Three different negative controls were used: no touch larvae, larvae injected with 0.85% NaCl and with heat-killed Cryptococci H99. Two positive controls of infected larvae were untreated and treated with empty PBCA-NP. n = 20 per group (*p* < 0.001). Effect of propolis-loaded PBCA-NP on *C. neoformans* fungal burden in mouse model.

To assess the antifungal activity of propolis-loaded PBCA-NP against *C. neoformans*, the survival of *G. mellonella* infected with H99 was studied. The results showed that our prepared PBCA-NP did not induce any toxicity on *G. mellonella* larvae as shown in Figure 9A.

The body of untreated and empty PBCA-NP-treated *G. mellonella* larvae became increasing darkened up to 80% death at 4 days after *C. neoformans* infection as shown in Figure 9B. No survivals were observed at 7 days post-infection in both groups. Interestingly, in the case of propolis-loaded PBCA-NP-treated *G. mellonella*, survival of larvae was increased approximately 90% on day 4. Treatment of propolis-loaded PBCA-NP rescued the infected larvae at 7 days post-infection. Overall survival studies of *C. neoformans* infection revealed that significantly prolonged (*p* < 0.001) in propolis-loaded PBCA-NP treatment than in empty PBCA-NP and untreated *G. mellonella* larvae groups. These results indicated that *C. neoformans* infection using an insect model could be cured by administering propolis-loaded PBCA-NP.

To determine the fungal burden in propolis-loaded PBCA-NP treatment and control mice on 8 days post-infection, enumerating CFUs in lung, kidney, and brain tissues was performed. Measurement of the mice body weight and determining the potential toxicity of propolis-loaded PBCA-NP was demonstrated as shown in Supplementary Figure S5–7.

**FIGURE 10:**
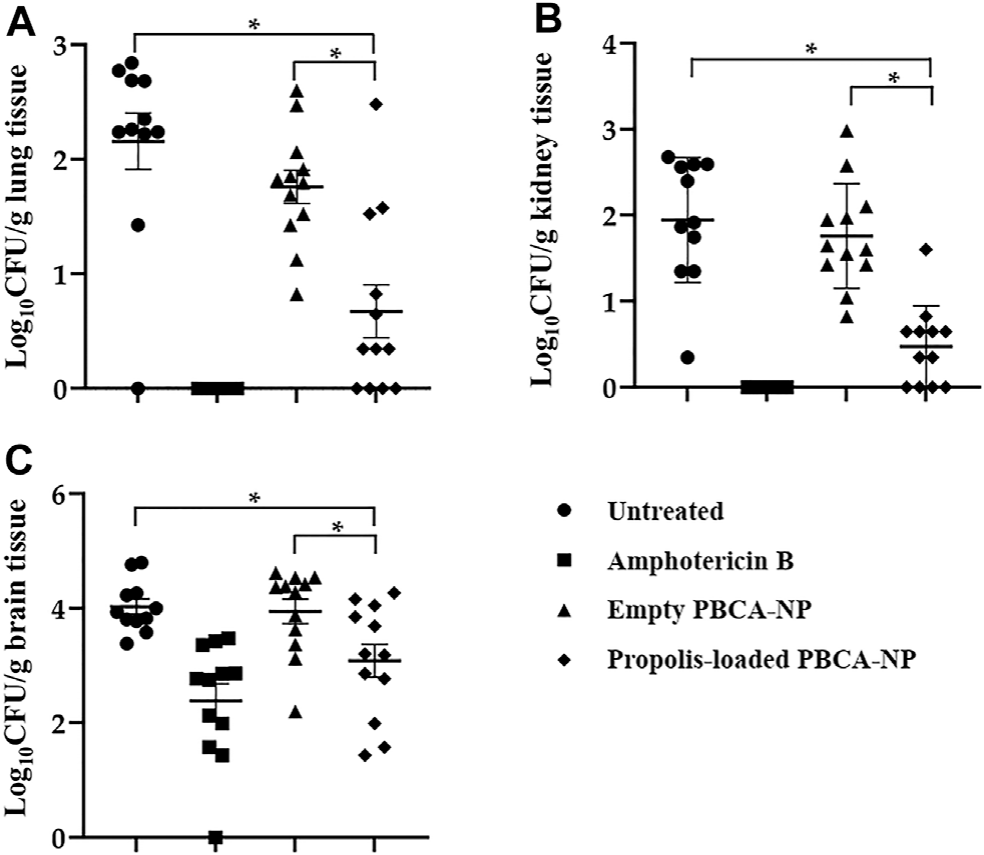
Fungal load in **(A)** lung, **(B)** kidney and **(C)** brain of Balb/c mice infected with *C. neoformans* treated or with untreated, amphotericin B, empty, or propolis-loaded PBCA-NP. n = 12 per group; “*” *p* ≤ 0.05.

The amount of *C. neoformans* remaining in organs was assessed and expressed as log_10_ (mean CFU/Gram tissue). There is no difference of fungal load in lung, kidney, and brain between untreated and empty PBCA-NP-treated mice as shown in Figure 10A–C. Propolis-loaded PBCA-NP treatment was clearly effective in reducing fungal loads in lung and kidney. Interestingly, fungal brain tissue in those treatment was dramatically decreased from 4.03 ± 0.45 (log_10_ CFU/g) in empty PBCA-NP-treated mice to 3.09 ± 0.99 (log_10_ CFU/g). Thus, we have demonstrated that propolis-loaded PBCA-NP led to greater decreases in fungal loads compared with empty PBCA-NP and untreated groups.

**FIGURE 11:**
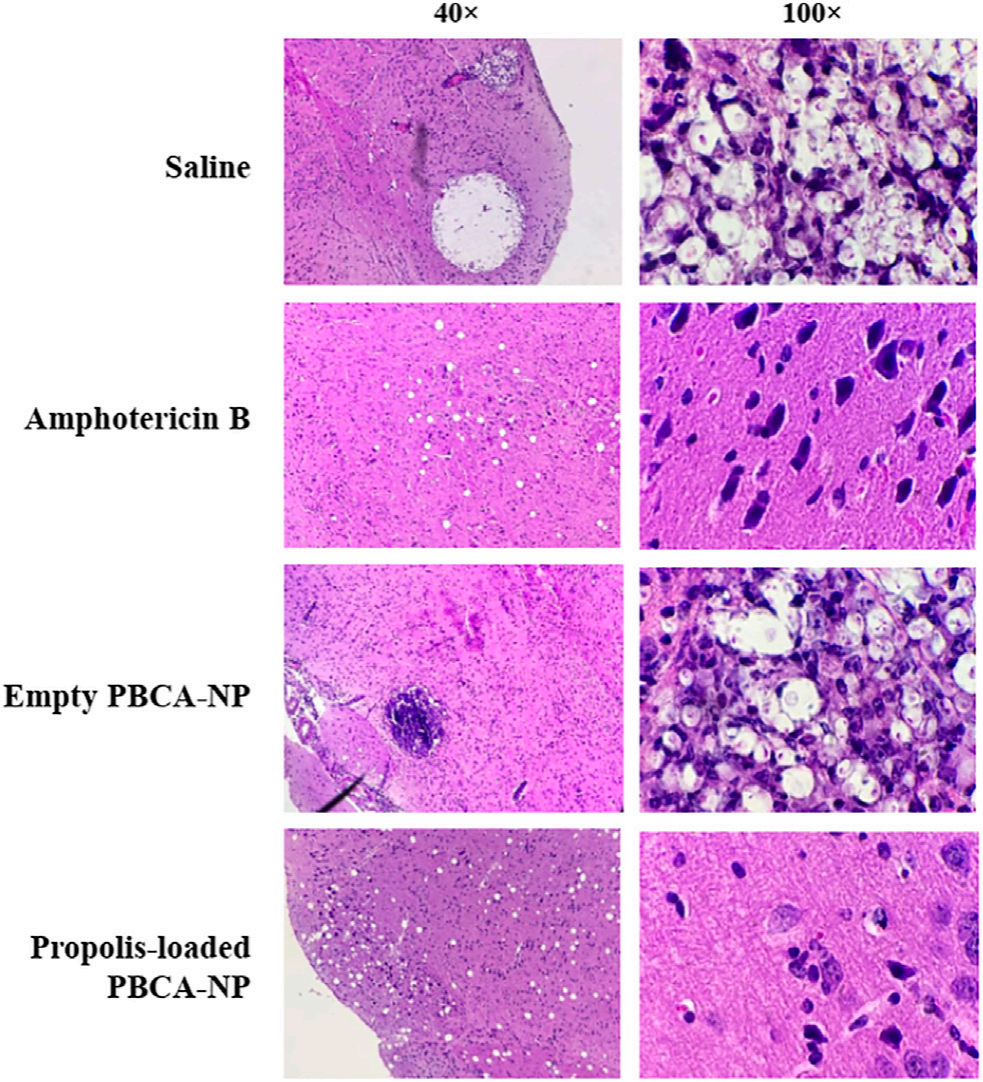
Representative histopathological sections of mouse brain tissue. Mice were humanely euthanized on days 8 post-inoculation and brain tissue was collected and stained with H&E. Sections were viewed by light microscopy at 40× and 100× magnification.

Histopathology revealed marked damage within the brain of mice inoculated with *C. neoformans*. Qualitatively, as evident by characteristic soap bubble cystic lesions and cryptococcal cells in the H&E-stained samples, clearly appeared in untreated and empty PBCA-NP-treated groups as shown in Figure 11. Interestingly, less histopathological lesions were seen in amphotericin B and propolis-loaded PBCA-NP-treated mice. Thus, intravenous injection of propolis-loaded PBCA-NP significantly reduced the fungal burden in brain tissue and the severity of histopathological changes. These results exhibited the therapeutically effect of propolis-loaded PBCA-NP in the *in vivo* model with cryptococcal infection.

## Discussion

Since propolis possesses very strong antimicrobial including antifungal activity, our basic idea in designing the propolis-loaded PBCA-NP mediated drug delivery system was to explore the possible effect of these prepared nanoparticles against the *C. neoformans* infection model. Our attempt is to maximize the efficacy of propolis through targeted nanoparticles, which also have the ability to kill *C.* *neoformans* in brain tissue. In this study, the results of physical characterization of propolis-loaded PBCA-NP were found to be satisfactory and effective, indicating an efficient drug delivery system. The prepared nanoparticles are spherical shape and less than 200 nm in size, with a negative surface charge, which is caused by carboxylic groups of the PBCA polymer. The negative zeta potential is responsible for electrostatic repulsion between particles (Feng and Huang, 2001). As expected, due to the presence of polysorbate 80 as stabilizer, the empty and propolis-loaded PBCA-NP were negatively charged with a zeta potential of −8.78 ± 1.24 mV. The uniformity of the nanoparticle dispersion was evaluated by the PDI. Values nearby to zero show a homogeneous dispersion, while those greater than 0.3 indicate high heterogeneity (Ahlin et al., 2002). The PDI of NP is of importance in controlling properties, nevertheless, the redispersing ability of the lyophilized suspendants of PBCA-NP on storage should be further investigated to ensure the uniformity after redispersion in the appropriate procedure (Xie et al., 2019). FTIR spectra of propolis-loaded PBCA-NP had similar absorbance patterns to that of propolis extract, indicating that the entrapment of propolis inside the nanoparticles was successful and propolis was not chemically modified by the encapsulation procedure.

Several factors contribute to the high entrapment efficiency of propolis (near 90%) into the PBCA-NP, including high solid-state solubility of drug/substance in the polymer, the solubility of drugs in the aqueous phase, and the fast rate of precipitation of the polymer in the organic phase. Additionally in this experiment, polymer–surfactant aggregates might be formed at higher surfactant concentrations minimizing the solubility of the drug into the aqueous phase (Xu et al., 2015). Studies of propolis release kinetics at different pH values in PBS, showed an initial burst release within the first 6 h, which may be associated with the distribution of a small amount of propolis from the surface of the PBCA-NP. The remaining propolis fraction is located inside the polymeric matrix and is released slowly over 24 h. These results are in accordance with reports that PBCA-NP exhibit a biphasic release pattern with a high initial burst release, followed by a slower controlled release of the drug from the particle (Kisich et al., 2007). Thus, propolis-loaded PBCA-NP can provide sustained release in the optimal environments.

*In vitro* BBB model was successfully generated and TEER values were used to indicate increased monolayer permeability. Our model showed the TEER values in primary cultured hCMEC/D3 cells are in the range of 30–50 Ω cm^2^ (Griep et al., 2013). Furthermore, a confluent hCMEC/D3 cell monolayer were grown on Transwell inserts and expresses the important tight junction structural protein (occludin) which further contributes to the control of tight junction integrity. Interestingly, the analysis of the percentage of the cellular translocation demonstrated that the PBCA-NP can pass through the hCMEC/D3 cell monolayer. These translocations can be further determined as BBB transport kinetics and represented in the apparent BBB permeability coefficient (P_app_) (Mészáros et al., 2018; Porkoláb et al., 2020). However, the insert membrane with 8 µm pore size may be too large and this may be the limitation of the system. Moreover, large pore size of insert membranes might let brain endothelial cells to form double layers and in the case of brain endothelial cell lines (bEnd.3), the tight junction formation on such membranes is disturbed (Wuest et al., 2013).

**FIGURE 12:**
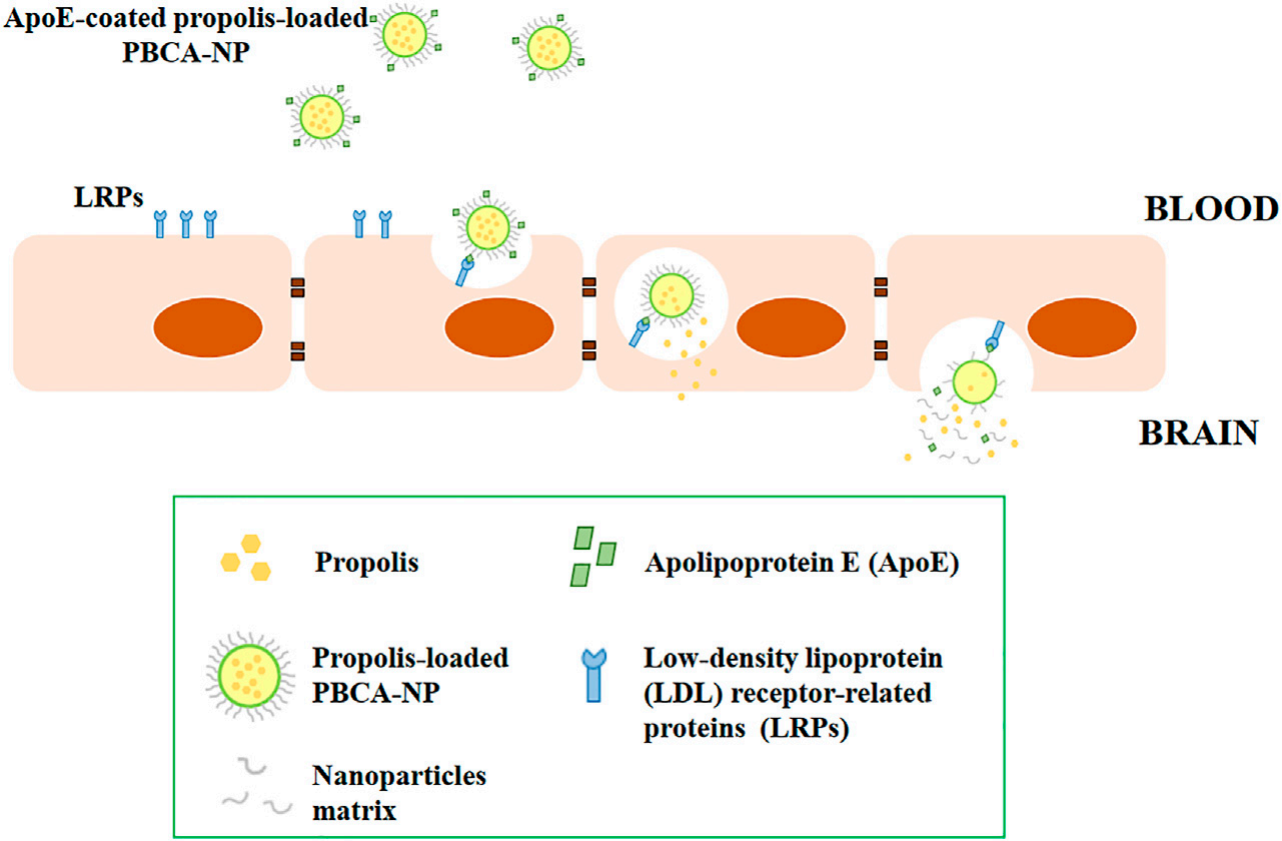
Schematic illustrations of the transport of ApoE-coated propolis-loaded PBCA-NP across the blood-brain barrier for drug delivery to the brain.

After the internalization of propolis-loaded or empty PBCA-NP into the hCMEC/D3 cells, these nanoparticles were observed by CLSM in the cell cytoplasm, particularly in lysosomes, where they would be presumably digested releasing propolis. The internalization mechanism of nanoparticles into the cell is through an endocytosis process (Sulheim et al., 2016). Some of the previous reports suggest that ApoE is adsorbed on the surface of nanoparticles coated with polysorbate 80, though the exact mechanism is not yet fully understood. Apolipoproteins attached to the surface of polysorbate 80-coated nanoparticles were found to facilitate the endocytosis process of endothelial cells within the brain parenchyma (Ramge et al., 2000). It has been reported that ApoE appears to play a vital role in the transport of low-density lipoprotein (LDL) into the brain, and non-ionic surfactant polysorbate 80 acts mainly as an anchor for the apolipoproteins-coated nanoparticles (Kreuter et al., 2002). Specific interaction to LDL receptor-related proteins (LRPs) was confirmed by utilization of the natural antagonist of LRP, receptor-associated protein (RAP) (Supplementary Figure S8). Therefore, the nanoparticles mimic low-density lipoproteins and specifically interact with the brain capillary endothelial cells, transferring their load into the brain *via* the LDL receptor-based endocytic mechanism as illustrated in Figure 12.

The nanomedicine approach in brain-infectious therapy mainly refers to the use of AmBisome, a liposomal formulation of amphotericin B. Several research methods using different animal models provide evidence of the effectiveness of AmBisome against brain infections, in which the inflammation, due to pathogens, alters the permeability of the BBB (Takemoto et al., 2006; Xu et al., 2011). Nevertheless, polymeric nanoparticles were recently investigated as an alternative treatment in brain-infectious diseases since *C. neoformans* has the unique ability to invade the CNS. Our prepared nanoparticles were first tested for *in vitro* activity against *C. neoformans*. In further experiments using an *in vitro* BBB model, our results highlighted that propolis-loaded PBCA-NP are enabled to pass through the BBB by LDL-receptor mediated endocytosis.

In this work, we establish the invertebrate *G. mellonella* as a suitable model for examining the toxicity and survival of larvae infected with *C. neoformans*. *G. mellonella* larvae have previously been used to study infection by various human fungal pathogens (Brennan et al., 2002; Schuhmann et al., 2003) and to study the pathogenicity of *C. neoformans* (Mylonakis et al., 2005; Fuchs et al., 2010). Our H99 strain killed the larvae with an effective inoculum of 2 × 10^6^ yeasts per larva. These inoculums are similar to those described previously for *C. neoformans* in *G. mellonella* (Fuchs et al., 2010). *In vivo* treatment of *G. mellonella* larvae infected with *C. neoformans* with propolis-loaded PBCA-NP showed that these nanoparticles significantly prolonged the survival of the infected larvae, an invertebrate host. This action can be attributed to the ability of propolis to inhibit the growth and virulence factors of cryptococcal cells as shown *in vitro* (Thammasit et al., 2018). Although testing in *G. mellonella* is unlikely to fully replace toxicity testing in mammals, it is a convenient step between *in vitro* tests and testing in mammals (Ignasiak and Maxwell, 2017), allowing to reduce to a minimum classical animal experiments.

Further study was performed in order to establish the safety and effectiveness of propolis-loaded PBCA-NP in the mouse model when given by intravenous route. The dose of PBCA-NP used had already been shown to be non-toxic in mice as it did not change in hematologic indices and blood biochemical levels (data not shown). The effect of antifungal treatment of propolis-loaded PBCA-NP was further established in term of fungal burden. Determination of fungal burden was clearly found the decrease in CFUs count of *C. neoformans* in the brain, the most affected organ (Khan et al., 2016). Animals treated with propolis-loaded PBCA-NP exhibited improved a reduction in the burden of *C. neoformans* in the brain after the termination of treatment. These might be due to; 1) The interference of cryptococcal viability and virulence factors in murine blood circulation, and 2) propolis-loaded PBCA-NP could potentially carry out multiple tasks; not only reduce the cryptococcal health, but also facilitate the delivery of propolis across the BBB. From our previous *in vitro* BBB model, propolis-loaded PBCA-NP obviously demonstrating that are able to pass through the BBB and belonging to the effect of cryptococcal killing, which is exactly at the ideal drug treatment in human situation. Thus, the importance in breaching the brain endothelial barrier and gaining access to the CNS of propolis-loaded PBCA-NP may increase the possibility of the use of biological administration as an alternative strategy for incurable diseases and can be addressed with an improvement in the functioning of nanotechnological drug delivery in the infectious treatment.

## Conclusion

We introduced the formulated PBCA-NP containing the natural honeybee product, propolis, that can be improved the solubility of propolis. PBCA-NP are not only biocompatible and biodegradable but also exhibit the bio-efficacy by *in vitro* and *in vivo* studies. The propolis-loaded PBCA-NP coated with polysorbate 80 and ApoE has shown great efficiency to pass through *in vitro* BBB model suggesting a highly promising targeting of brain infections. Additionally, invertebrate *G. mellonella* larvae and mouse infection models have been used to demonstrate anti-fungal potential of propolis loaded PBCA-NP towards *C. neoformans* infection. To the best of our knowledge, this is the first report to represent the efficacy of propolis in the cerebral cryptococcosis models. Therefore, our propolis loaded PBCA-NP promise a higher performance to target brain infection areas and represent an advantageous alternative to similar formulations of conventional drugs avoiding their serious side effects.

## Data Availability

The original contributions presented in the study are included in the article/Supplementary Material, further inquiries can be directed to the corresponding author.
