# Supplementary material for: Targeted Propolis-Loaded Poly (Butyl) Cyanoacrylate Nanoparticles: An Alternative Drug Delivery Tool for the Treatment of Cryptococcal Meningitis
Source: Front Pharmacol. 2021 Aug 20;12:723727. doi: 10.3389/fphar.2021.723727 (PMC8417799; doi:10.3389/fphar.2021.723727)
Supplement: Supplementary file 1 [file DataSheet1.docx]

Supplementary Material

Propolis was firstly diluted and scanned the absorbance spectra (190–600 nm) and further determined the absorbance maximum (λmax). A UV absorbance maximum peak at 267 nm is attributed to major polyphenol compounds including phenolic compounds and flavonoids as the active components in propolis as shown in Supplementary Figure 1.


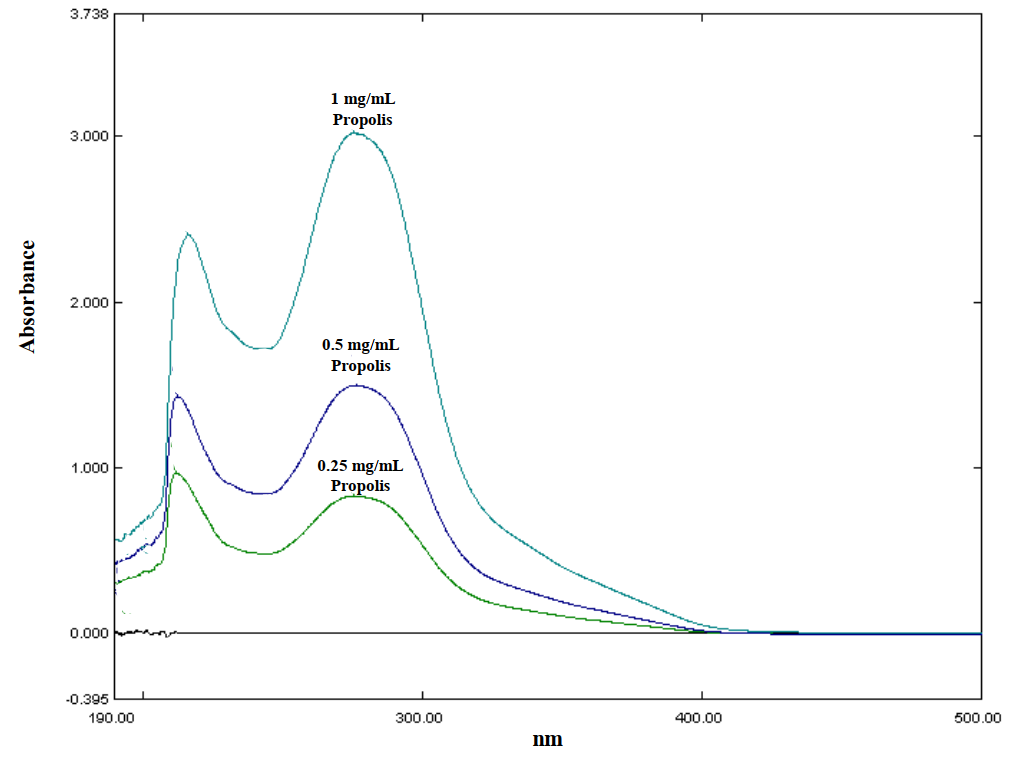


**Supplementary Figure 1** UV-Vis spectra of propolis extract.

The viability of 10^5^ hCMEC/D3 cells treated with 5 concentrations (10^5^-10^11^ particles) of propolis-loaded and empty PBCA-NP was detected by the MTT assay. The results showed the cell survival rates were over 90%. This suggested that propolis-loaded and empty PBCA-NP enter the hCMEC/D3 cell and are non-toxic to cellular function as shown in Supplementary Figure 2.

.


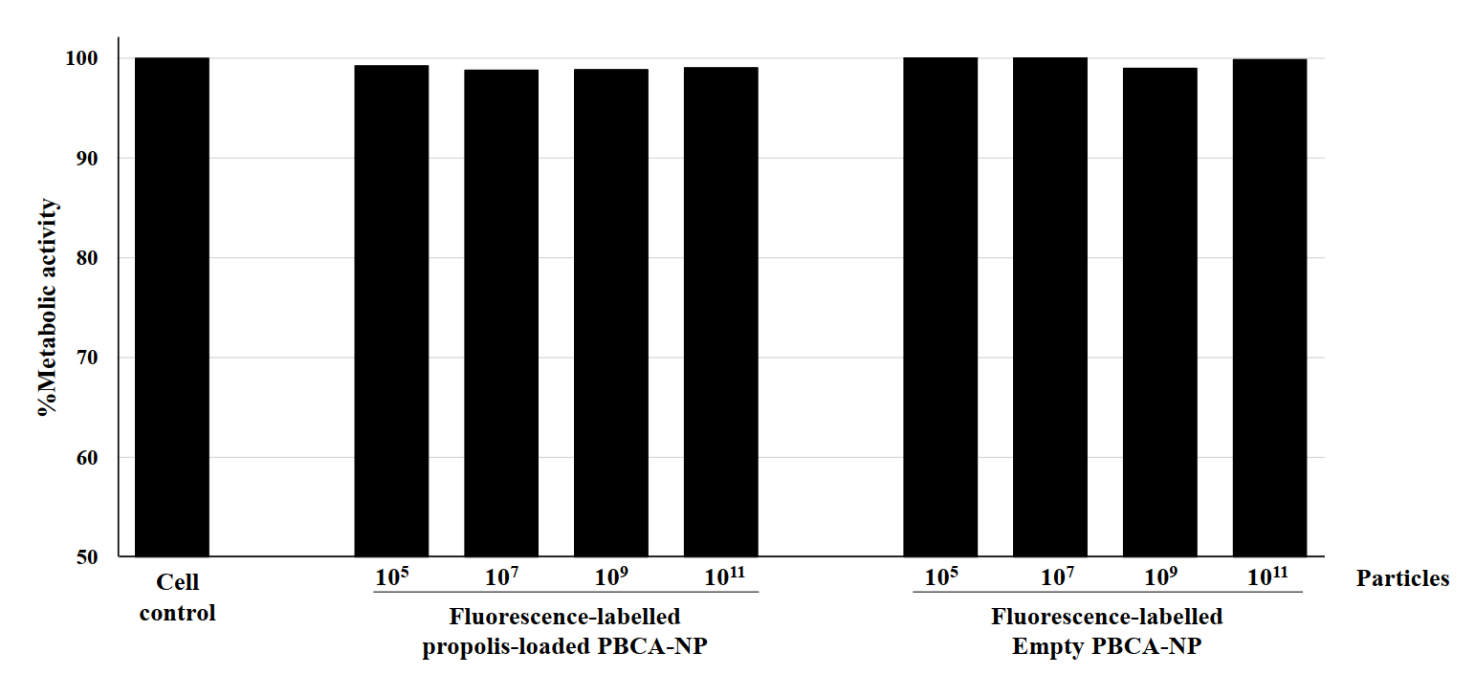


**Supplementary Figure 2.** Percentage metabolic activity of hCMEC/D3 cells upon exposure to fluorescence-labelled propolis-loaded and empty PBCA-NP.

After reaching confluency, hCMEC/D3 cells were loaded with propolis-loaded and harvested by trypsinization. Subsequently, images were acquired using confocal laser scanning microscope (CLSM) as shown in Supplementary Figure 3. Lysosomes were visualized by LysoTracker Green (Figure 3A) and Nile red-labelled propolis-loaded PBCA-NPs were uptaken and distributed throughout cytoplasmic compartment (Figure 3B). The merged yellow color confirmed that the PBCA-NP localized exclusively at lysosomes in the cytoplasm compartment (Figure 3C and 3D). In addition, the uptake percentage of cells incubated with ApoE coated propolis-loaded PBCA-NP was clearly higher than that of uncoated PBCA-NP, suggesting that conjugation of ApoE could improve binding and uptake of propolis-loaded PBCA-NP to hCMEC/D3.

*
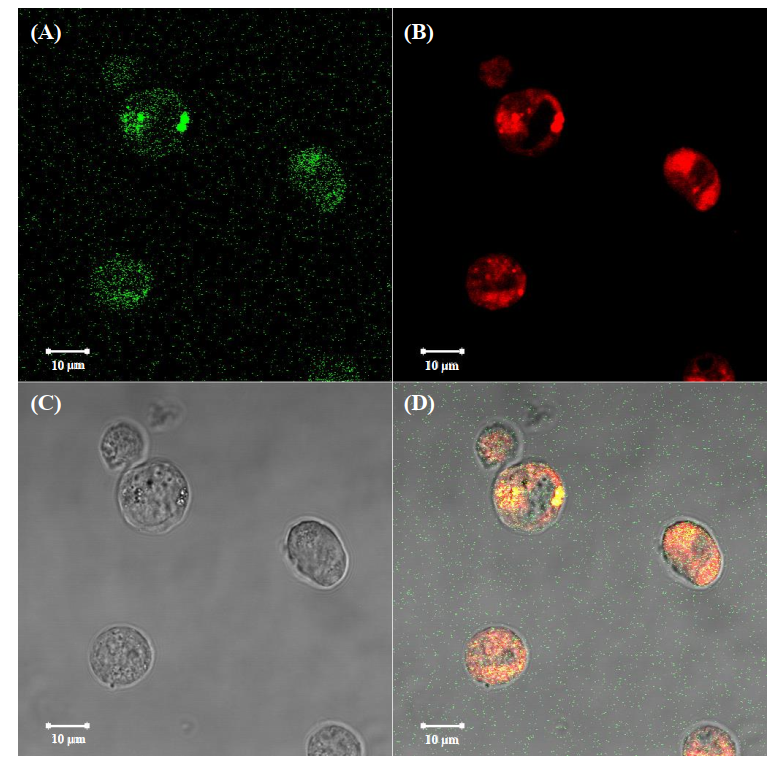
*

**Supplementary Figure 3.** CLSM images of trypsinized hCMEC/D3 cells represented the intracellular localization of ApoE coated propolis-loaded PBCA-NP. (A) Lysosomes were stained with LysoTracker Green (green), (B) propolis-loaded PBCA-NP were labelled with Nile red (red), (C) DIC image, and (D) colocalization of puncta with propolis-loaded PBCA-NP and lysosomes (yellow). Scale bars represent 10 µm.

The presence of mature occludin tight junctions in the cell monolayer was also observed and images were acquired with the 25× objective on a Zeiss CellDiscoverer 7 (Carl Zeiss Microscopy, Oberkochen, Germany) as shown in Supplementary Figure 4.


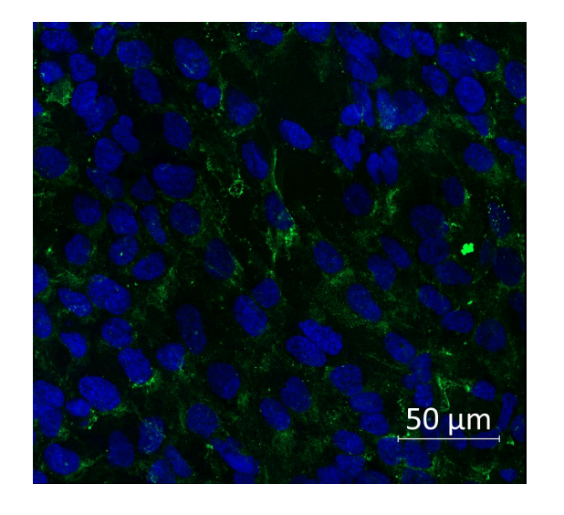


**Supplementary Figure 4.** Immunofluorescent staining of tight junction proteins occludin in hCMEC/D3 BBB model.

The effect of propolis-loaded PBCA-NP on *C. neoformans*-infected mice was firstly determined in the mice body weight. As shown in Supplementary Figure 5, the results displayed slightly decreased along with a disease progression. In addition, the potential toxicity of propolis-loaded PBCA-NP was investigated by blood profile determination. The results represented that the administration of propolis-loaded or empty PBCA-NP had no direct adverse effect on blood indicies (Supplementary Figure 6) and kidney function (Supplementary Figure 7) in *C. neoformans*-infected mice.


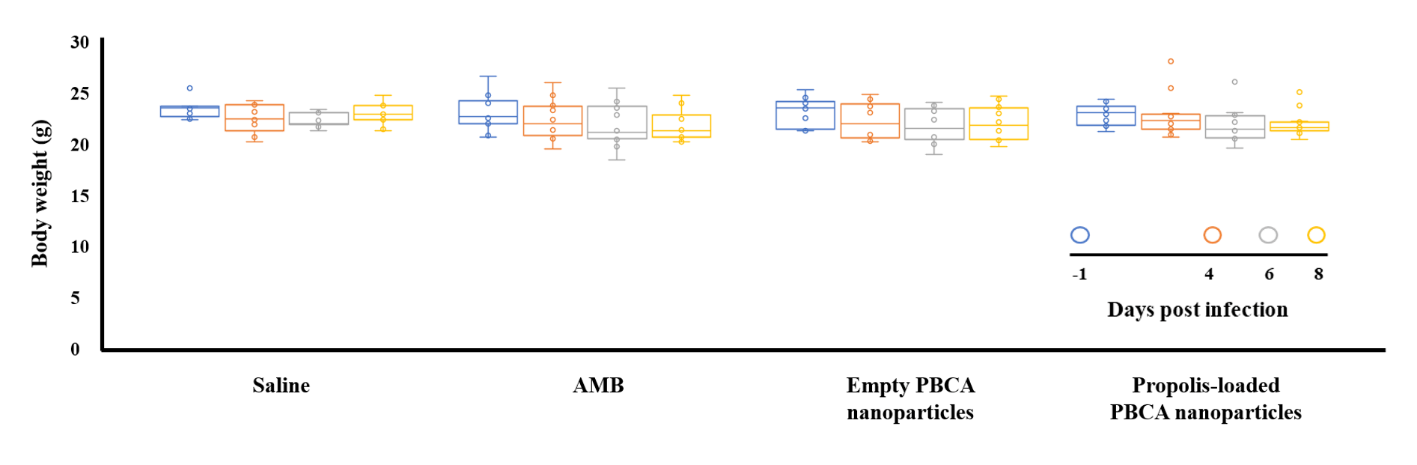


**Supplementary Figure 5.** Measurement of murine body weight after cryptococcal infection.


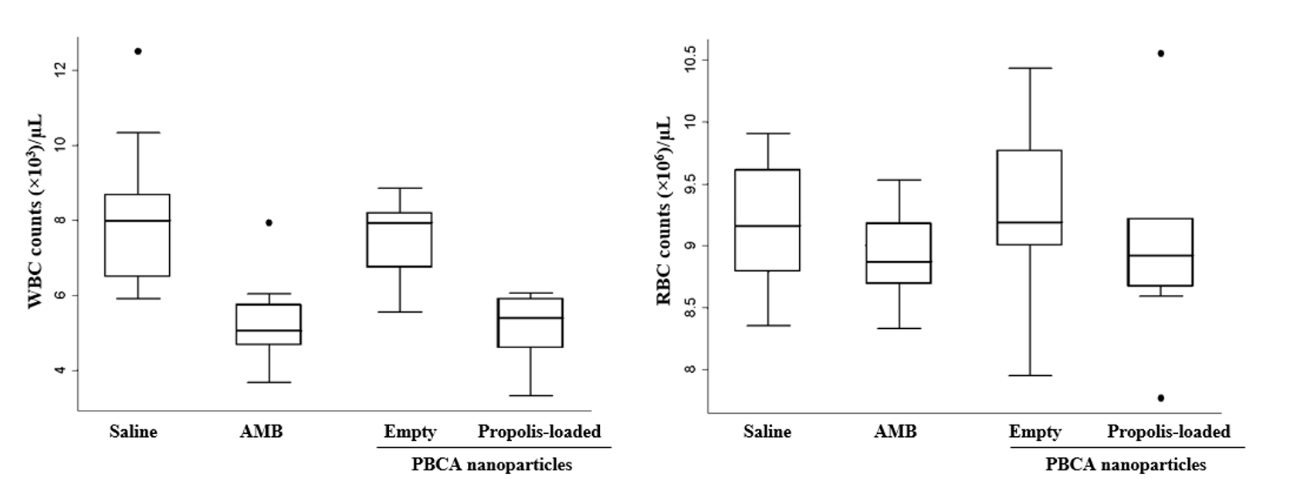


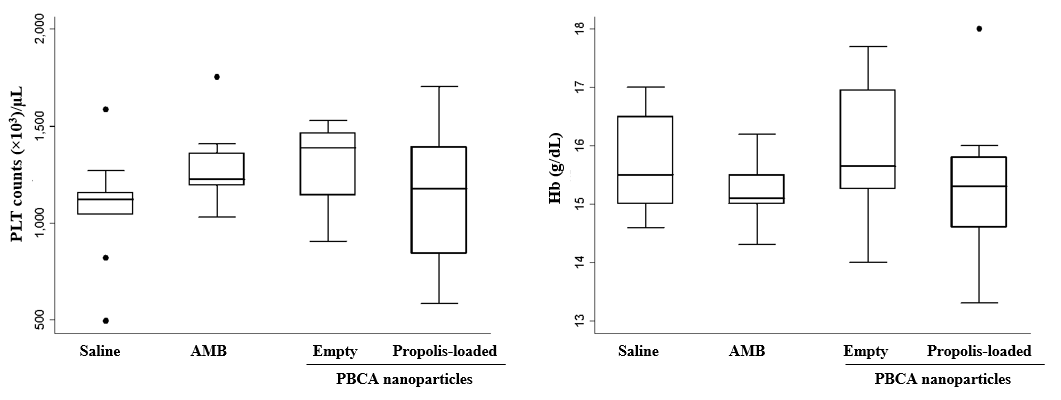


**Supplementary Figure 6.** Whole-blood analysis after PBCA NP treatment.


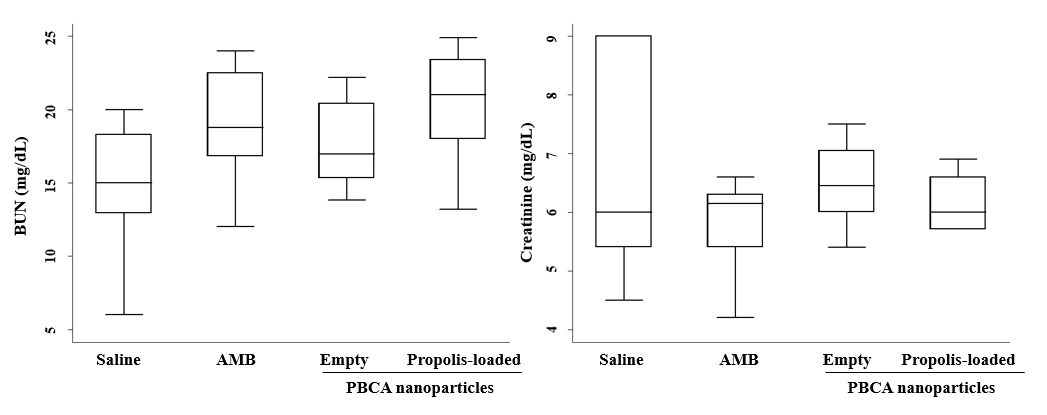
 **Supplementary Figure 7.** Mouse sera levels of blood urea nitrogen and creatinine as an indicator of kidney function.

The hCMEC/D3 cells were incubated with receptor-associated protein (RAP) antagonist, an inhibitor of low-density lipoprotein (LDL) receptor-mediated endocytosis, in order to investigate propolis-loaded PBCA-NP uptake pathways. The cells were incubated with nile red-labelled propolis-loaded and empty PBCA-NP in the presence or absence of 500 nM and 1 µM of RAP endocytosis inhibitors. Both of nile red-labelled PBCA-NP transcytosis were decreased in the presence of RAP for 24h incubation as shown in Supplementary Figure 8. Thus, LDL receptor mediated endocytosis of propolis-loaded PBCA-NP was inhibited by RAP especially in the pre-treatment with 1 µM of RAP.


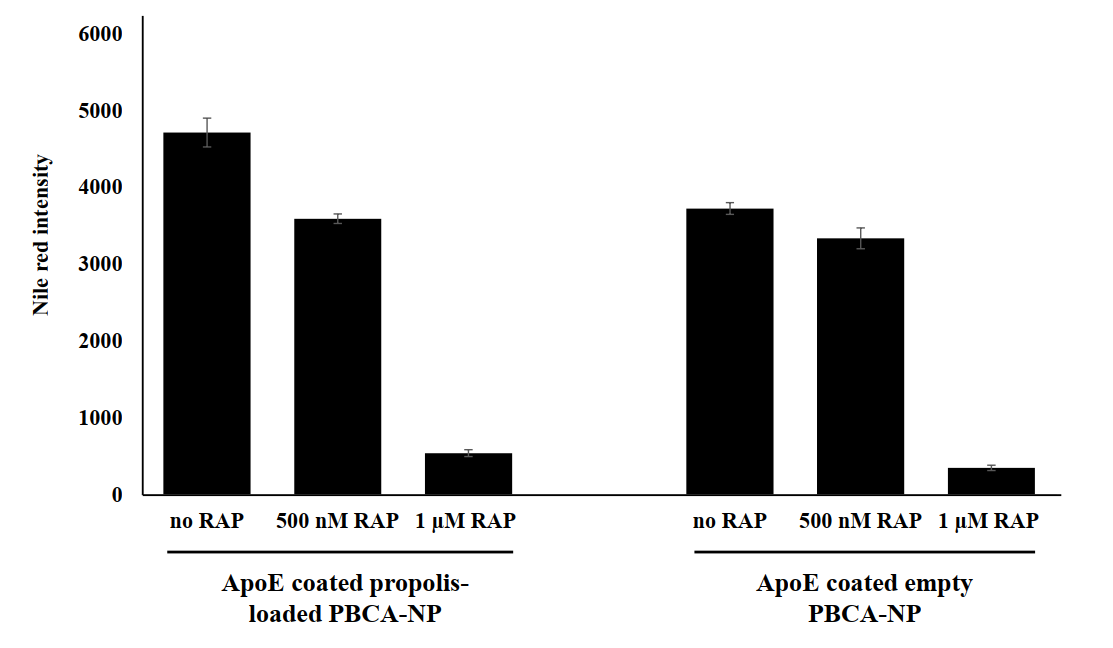


**Supplementary Figure 8.** Transcytosis of nile red-labelled PBCA-NP in the *in vitro* BBB model after 24 h of exposure to the nanoparticles in the presence and absence of 500 nM and 1 µM of RAP.
